# Recurrent Intensive Care Episodes and Mortality Among Children With Severe Neurologic Impairment

**DOI:** 10.1001/jamanetworkopen.2024.1852

**Published:** 2024-03-15

**Authors:** Katherine E. Nelson, Jingqin Zhu, Joanna Thomson, Sanjay Mahant, Kimberley Widger, Chris Feudtner, Eyal Cohen, Eleanor Pullenayegum, James A. Feinstein

**Affiliations:** 1 Pediatric Advanced Care Team, Hospital for Sick Children, Toronto, Ontario, Canada; 2 Division of Paediatric Medicine, Department of Paediatrics, Hospital for Sick Children, Toronto, Ontario, Canada; 3 Child Health Evaluative Sciences, SickKids Research Institute, Toronto, Ontario, Canada; 4 ICES, Toronto, Ontario, Canada; 5 Institute for Health Policy, Management and Evaluation, Dalla Lana School of Public Health, University of Toronto, Toronto, Ontario, Canada; 6 Department of Pediatrics, University of Cincinnati College of Medicine, Cincinnati, Ohio; 7 Division of Hospital Medicine, Cincinnati Children’s Hospital Medical Center, Cincinnati, Ohio; 8 CanChild Centre for Childhood Disability Research, McMaster University, Hamilton, Ontario, Canada; 9 Lawrence S. Bloomberg Faculty of Nursing, University of Toronto, Toronto, Ontario, Canada; 10 The Justin Michael Ingerman Center for Palliative Care, Children’s Hospital of Philadelphia, Philadelphia, Pennsylvania; 11 Department of Pediatrics, Perelman School of Medicine, University of Pennsylvania, Philadelphia; 12 Department of Medical Ethics and Health Policy, Perelman School of Medicine, University of Pennsylvania, Philadelphia; 13 Edwin S.H. Leong Centre for Healthy Children, University of Toronto, Toronto, Ontario, Canada; 14 Adult and Child Consortium for Health Outcomes Research and Delivery Science, University of Colorado and Children’s Hospital Colorado

## Abstract

**Importance:**

Children requiring care in a pediatric intensive care unit (PICU) are known to have increased risk of subsequent mortality. Children with severe neurologic impairment (SNI)—who carry neurologic or genetic diagnoses with functional impairments and medical complexity—are frequently admitted to PICUs. Although recurrent PICU critical illness episodes (PICU-CIEs) are assumed to indicate a poor prognosis, the association between recurrent PICU-CIEs and mortality in this patient population is poorly understood.

**Objective:**

To assess the association between number of recent PICU-CIEs and survival among children with severe neurologic impairment.

**Design, Setting, and Participants:**

This population-based retrospective cohort study used health administrative data from April 1, 2002, to March 31, 2020, on 4774 children born between 2002 and 2019 with an SNI diagnosis code in an Ontario, Canada, hospital record before 16 years of age and a first PICU-CIE from 2002 to 2019. Data were analyzed from November 2021 to June 2023.

**Exposure:**

Pediatric intensive care unit critical illness episodes (excluding brief postoperative PICU admissions).

**Main Outcome and Measures:**

One-year survival conditioned on the number and severity (length of stay >15 days or use of invasive mechanical ventilation) of PICU-CIEs in the preceding year.

**Results:**

In Ontario, 4774 children with SNI (mean [SD] age, 2.1 [3.6] months; 2636 [55.2%] <1 year of age; 2613 boys [54.7%]) were discharged alive between 2002 and 2019 after their first PICU-CIE. Ten-year survival after the initial episode was 81% (95% CI, 79%-82%) for children younger than 1 year of age and 84% (95% CI, 82%-86%) for children 1 year of age or older; the age-stratified curves converged by 15 years after the initial episode at 79% survival (95% CI, 78%-81% for children <1 year and 95% CI, 75%-84% for children ≥1 year). Adjusted for age category and demographic factors, the presence of nonneurologic complex chronic conditions (adjusted hazard ratio [AHR], 1.70 [95% CI, 1.43-2.02]) and medical technology assistance (AHR, 2.32 [95% CI, 1.92-2.81]) were associated with increased mortality. Conditional 1-year mortality was less than 20% regardless of number or severity of recent PICU-CIEs. Among children with high-risk PICU-CIEs, 1-year conditional survival decreased from 90% (95% CI, 89%-91%) after the first PICU-CIE to 81% (95% CI, 77%-86%) after the fourth PICU-CIE.

**Conclusions and Relevance:**

This cohort study of children with SNI demonstrated a modest dose-dependent association between PICU-CIEs and short-term mortality. These data did not support the conventional wisdom that recurrent PICU admissions are associated with subsequent high mortality risk.

## Key Points

**Question:**

Are the number and severity of recent critical illness episodes associated with increased short-term mortality among children with severe neurologic impairment (SNI) admitted to the pediatric intensive care unit (PICU)?

**Findings:**

In this cohort study comprising 4774 children with SNI admitted to the PICU, survival at 15 years after their initial episode was 79%. One-year survival after high-risk episodes decreased from 90% for the first episode to 81% after the fourth episode.

**Meaning:**

This study suggests that there is a modest dose-dependent association between number of recent high-risk critical illness episodes and mortality among children with SNI.

## Teaser

This cohort study assesses the association between number of recent pediatric intensive care unit (PICU) critical illness episodes and survival among children with severe neurologic impairment.

## Introduction

Children who are discharged alive from the pediatric intensive care unit (PICU) have an increased risk of mortality, approximately 5% over 5 years for the general PICU population.^1^ Among children with complex chronic conditions (CCCs), the risk of mortality is 8% over 4 years, increasing to 17% over 4 years among those with recurrent PICU admissions^2^ for chronic critical illness.^3,4,5^

A subset of children with CCCs have severe neurologic impairment (SNI) with underlying neurologic or genetic diagnoses that are associated with significant functional impairments and medical complexity.^6^ Although they comprise only about 0.2% of children,^7^ those with SNI accrue approximately 25% of PICU admissions,^8^ are more likely to be readmitted than children with other chronic conditions,^9^ and are at increased risk of respiratory failure from acute respiratory distress syndrome.^10^ For caregivers of children with SNI, end-of-life decision-making is common; in a survey of 103 bereaved parents, 42% reported decisions to withhold or withdraw life-sustaining therapies.^11^ Likelihood of survival is an important factor in these decisions,^12^ and patterns of PICU admissions are included in the estimation of mortality risk. A common clinical belief is that increasing frequency of PICU admissions represents a health trajectory portending death. However, to our knowledge, the association between recurrent PICU admissions and subsequent mortality for children with SNI has not been quantitatively evaluated.

We assessed survival after critical illness episodes (CIEs) among children with SNI born from 2002 to 2019 in Ontario, Canada. Specifically, we tested the hypothesis that more frequent CIEs in the preceding year would be associated with higher 1-year mortality.

## Methods

### Study Design and Data Sources

This population-based retrospective cohort study used Ontario health administrative data, which included all children and hospitals in the province. The most populated Canadian province, Ontario has 14.6 million residents who receive all acute care through a single-payer public health care system. We used linked health administrative databases available at ICES to identify and follow up data over time on Ontario children with SNI. ICES is an independent, nonprofit research institute with special status under Ontario’s Personal Health Information Protection Act (PHIPA), which allows researchers to access health and demographic data to evaluate and improve the health care system without individual consent. This project’s use of data was authorized under section 45 of PHIPA, approved by ICES’ Privacy and Legal Office, and did not require additional review by a research ethics board. We accessed health administrative records (Canadian Institute for Health Information Discharge Abstract Database and the Ontario Health Insurance Plan database) and demographic data (Registered Persons Database). Datasets were linked using unique encoded identifiers. We followed the Strengthening the Reporting of Observational Studies in Epidemiology (STROBE) reporting guideline for cohort studies.^13^

### Cohort Creation

We constructed the cohort of children with SNI using a clinically validated list of *International Classification of Diseases and Related Health Problems, Tenth Revision, Canadian version* (*ICD-10-CA*) codes (eAppendix 1 in Supplement 1).^14^ All children who had an SNI diagnosis code in an Ontario hospitalization record prior to their 16th birthday and a date of birth between April 1, 2002, and March 31, 2019, were included in the cohort. To exclude children who arrived in Ontario with advanced disease, children born outside of Ontario must have been eligible for provincial insurance for 1 year prior to the first hospitalization with an SNI diagnosis. In addition, children who left the province within 1 year of their initial SNI diagnosis were excluded for inadequate follow-up time. All data are reported by fiscal year (April 1 to March 31). Because of pandemic-associated changes in health care use, we ended study follow-up on March 31, 2020, which includes approximately 2 weeks of COVID-19 era data.

### Critical Illness Episodes

Because of our interest in the accuracy of mortality prognosis, we focused on PICU admissions associated with critical illness (eAppendix 2 in Supplement 1). Children with SNI are at increased risk for anesthesia complications, so routine postprocedural monitoring in the ICU is common^8^ and is unlikely to be considered a factor associated with mortality by a clinician estimating mortality risk. Therefore, when identifying CIEs, we excluded PICU stays occurring within 24 hours of a procedure requiring anesthesia (eg, surgery) (eAppendix 2 in Supplement 1) and lasting less than 48 hours. We did not exclude longer postoperative PICU admissions because they could reflect the surgery’s significance or postoperative complications; both would likely influence mortality risk assessment. All other PICU admissions during the study period were considered CIEs and were included in the analysis. We did not set age-based limits, but PICU admissions among individuals older than 18 years of age are rare in Ontario.

### Descriptive Analyses

We identified children’s SNI diagnostic categories^15^ based on the SNI codes in the hospitalization record for the initial CIE. Among children without SNI codes in the hospitalization record for their initial CIE, we derived the SNI diagnostic category from their first SNI hospitalization. Area-level indicators of rurality and socioeconomic status were derived from census data using the postal code listed in the hospitalization record for the initial CIE. We defined rural or urban residence using the Rurality Index of Ontario,^16^ which assesses population density and health care accessibility. Income quintiles were assigned based on population averages from the census dissemination area (400-700 people). Sex was obtained from the administrative dataset Registered Persons Database. Details about race and ethnicity are not available in the data sources and thus were not assessed. We defined comorbidities according to *ICD-10-CA* codes identified as CCCs,^17^ omitting *ICD-10-CA* codes that were cross-listed on the SNI diagnosis list. Similarly, we used a list of *ICD-10-CA* codes describing medical technology assistance that were developed by the Canadian Institute for Health Information specifically for use in categorizing children with medical complexity.^7^ Because medical technology assistance and CCCs are inconsistently recorded in hospitalization records, we included a lookback to the initial hospitalization with an SNI diagnosis to improve sensitivity in their identification.^7^ We also described features of the CIEs, including the number of episodes per child, the frequency of high-risk markers, and the time to event from first to second and from second to third CIE.

### Survival

Using death data from the Registered Persons Database, which captures all deaths reported to the province irrespective of location (hospital, home, hospice, or other facility), we calculated survival from the date of PICU discharge until date of death. Surviving children’s data were censored at study end (March 31, 2020) or if they moved out of Ontario (eAppendix 2 in Supplement 1).

### Statistical Analysis

#### Baseline Long-Term Survival

Statistical analysis was conducted from November 2021 to June 2023, using SAS Enterprise, version 7.1 (SAS Institute Inc). All *P* values were from 2-sided tests, and results were deemed statistically significant at *P* < .05. Our primary analysis was designed to test the general assumption that the recent frequency of CIEs is associated with mortality, irrespective of individual-level characteristics. Conditional survival estimates, however, require a base case for comparative interpretation, so we calculated long-term survival estimates for the cohort from the date of first CIE discharge in Ontario after or concurrent with first SNI diagnosis. Because the baseline risk of childhood mortality is highest during the first year of life,^18^ we chose a priori to stratify by age (<1 year and ≥1 year). To understand the association of individual-level factors with long-term survival, we created age-stratified Cox proportional hazards regression models from discharge date of initial PICU-CIE. Our Cox proportional hazards regression models included baseline demographic characteristics defined at the time of initial PICU-CIE: year, sex, rurality, income quintile, presence of non-SNI–associated CCCs, and use of medical technology assistance. Because many children had multiple SNI and non-SNI complex CCCs and diagnoses were inconsistently recorded across episodes, we did not include diagnostic categories in models.

#### Short-Term Survival Conditioned on Number of Recent Episodes

To assess the strength of the association between number of recent PICU-CIEs and short-term mortality, the analysis was anchored at the level of the episode (ie, a child with multiple episodes contributed data for each episode). However, not all PICU-CIEs are likely associated with the same risk of mortality. For example, some children with SNI require intensive care to support their baseline medical technology. To identify PICU admissions that more likely indicate worsening of a child’s baseline health, we stratified the short-term survival analyses by risk (standard risk vs high risk). We defined high-risk admissions as those with increased risk of mortality based on either prolonged stay^19^ or illness severity. In prior studies of PICU admissions for children with CCCs, prolonged length of stay was defined as more than 15 days.^20^ We chose intubation as the marker of illness severity because invasive mechanical ventilation is a risk factor for mortality^21^ and a marker of illness severity on the Pediatric Index of Mortality.^22^ For children with chronic ventilatory needs, we considered only prolonged admissions to be high risk; their other episodes were classified as standard risk.

To understand the association between CIEs and subsequent mortality, our primary analysis estimated 1-year survival conditioned on the number of high-risk episodes in the preceding year. For each CIE, we looked back 1 year from the date of PICU admission to assess the number and risk of preceding episodes in the preceding year. We did not differentiate between initial admissions and unplanned readmissions; a child could accrue multiple PICU-CIEs within a single hospitalization. However, if more than 1 year elapsed between 2 PICU-CIEs, both would contribute to the analysis for the first discharge. We assumed that increased mortality risk would persist 1 or more years after a high-risk episode. Thus, an episode was classified as high risk if it or any of the preceding episodes in the prior year were high risk (length of stay >15 days or use of invasive mechanical ventilation); all other episodes were standard risk. Within the high-risk and standard-risk categories, we assessed change in survival conditioned on number of previous episodes.

## Results

### Children With SNI and CIEs

**Figure 1.**
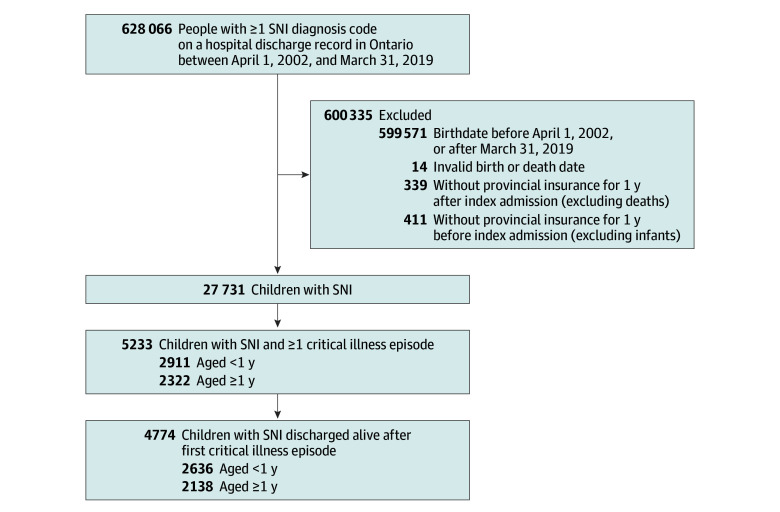
Cohort Creation For this study, we excluded brief postprocedural critical care unit admissions occurring within 24 hours of a surgical procedure and lasting less than 48 hours. SNI indicates severe neurologic impairment.

In Ontario, 4774 children with SNI were discharged alive after their first PICU-CIE between 2002 and 2019; most were male (2613 boys [54.7%] and 2161 girls [45.3%]) and younger than 1 year of age (2636 [55.2%]), with a mean (SD) age of 2.1 (3.6) months (Table 1). They represented 17.2% of the 27 731 children in Ontario with SNI-associated diagnosis codes in a hospital discharge record from 2002 to 2019 (Figure 1). Most of the cohort (3050 [63.9%]) had additional CCCs not associated with SNI; many children (1240 [26.0%]) had CCCs affecting 2 or more organ systems (Table 1). Approximately 40% of the cohort (1893 [39.7%]) had a cardiac CCC. Among children with 2 or more CIEs (n = 1728), the median time from the first to second episode was 2.5 months (IQR, 0.3-11.3 months). For children with 3 or more episodes (n = 864), the median time from the second to third episode was 3.1 months (IQR, 0.6-11.9 months). Most individuals accrued 1 or 2 CIEs during childhood (3910 [81.9%]); 306 children (6.4%) had 5 or more episodes. Among all CIEs (n = 8995), 4158 episodes (46.2%) included invasive mechanical ventilation, 1267 episodes (14.1%) lasted more than 15 days, and 4552 episodes (50.6%) included 1 or more severity markers.

**Table 1.** Clinical Characteristics of Children With Severe Neurologic Impairment Discharged Alive After Their First Critical Illness Episode

Characteristic	Children, No. (%) (N = 4774)
Year of first critical illness episode	
2002-2006	856 (17.9)
2007-2011	1292 (27.1)
2012-2016	1390 (29.1)
2017-2019	1236 (25.9)
Age at first critical illness episode discharge, y	
<1	2636 (55.2)
1 to <2	501 (10.5)
2 to <5	731 (15.3)
≥5	906 (19.0)
Sex	
Female	2161 (45.3)
Male	2613 (54.7)
Rural residence	
No	4371 (91.4)
Yes	323 (6.8)
Missing	80 (1.7)
Neighborhood income quintile	
First (lowest)	1201 (25.2)
Second	939 (19.7)
Third	968 (20.3)
Fourth	909 (19.0)
Fifth	722 (15.1)
Missing	35 (0.7)
SNI diagnostic categories^a^	
Anatomic	1202 (25.2)
Genetic	1075 (22.5)
Epilepsy	794 (16.6)
Static	667 (14.0)
Peripheral	616 (12.9)
Stroke or hemorrhage	433 (9.1)
Progressive movement	182 (3.8)
Metabolic	134 (2.8)
No. of chronic medical technologies	
None	1554 (32.6)
1	1371 (28.7)
2	967 (20.0)
≥3	882 (18.5)
No. of organ systems affected by additional nonneurologic complex chronic conditions	
None	1724 (36.1)
1	1810 (37.9)
2	824 (17.3)
≥3	416 (8.7)
Type of nonneurologic complex chronic condition^a^	
Cardiac	1893 (39.7)
Other congenital or genetic	664 (13.9)
Metabolic	436 (9.1)
Respiratory	429 (9.0)
Urologic or kidney	385 (8.1)
Gastrointestinal	371 (7.8)
Hematologic or immunologic	344 (7.2)
Oncologic	337 (7.1)
Medical devices and transplant	<6 (<0.1)

Abbreviation: SNI, severe neurologic impairment.

^a^ Children may be included in multiple categories.

### Long-Term Survival From Initial PICU Discharge

**Figure 2.**
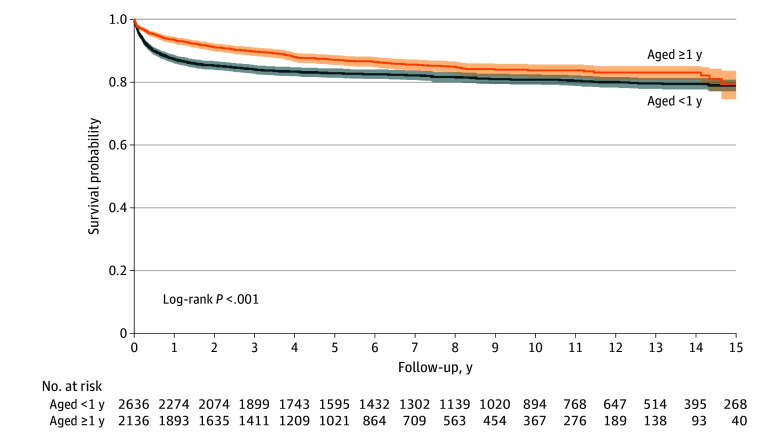
Long-Term Survival of Children With Severe Neurologic Impairment After Their First Critical Illness Episode Survival curves are stratified by age as younger than 1 year and 1 year or older.

**Figure 3.**
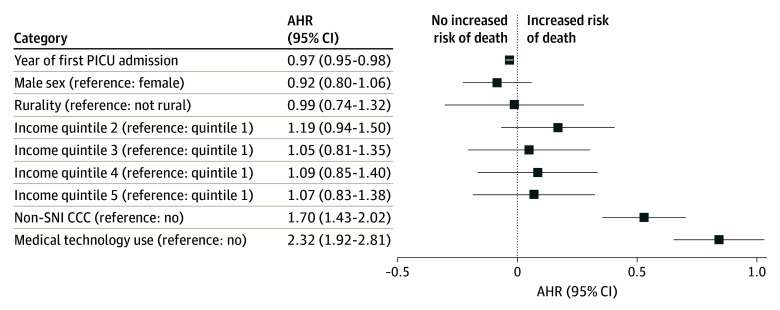
Factors Associated With Mortality After Children’s First Critical Illness Episodes This forest plot does not show missing categories for rurality or income quintile (≤2%). AHR indicates adjusted hazard ratio; CCC, complex chronic condition; PICU, pediatric intensive care unit; and SNI, severe neurologic impairment.

Among the 4774 children discharged alive, long-term survival after initial PICU discharge was lower among children younger than 1 year of age at discharge compared with those 1 year of age or older (81% [95% CI, 79%-82%] vs 84% [95% CI, 82%-86%]; log-rank *P* < .001) (Figure 2). By 15 years after discharge, the survival curves converged at 79% (<1 year: 95% CI, 78%-81%; ≥1 year: 95% CI, 75%-84%). Much of the mortality risk accrued during the first 24 months after discharge; 2-year survival was 85% (95% CI, 84%-87%) for children younger than 1 year of age and 91% (95% CI, 79%-82%) for children 1 year of age or older (eFigure 1 in Supplement 1). In an age-stratified Cox proportional hazards regression model, the shapes of the hazard function varied, but the coefficients were the same in both strata. Adjusting for demographic characteristics (year of admission, sex, rurality, and income quintile), there was an increased risk of death for children who had long-term medical technology use (adjusted hazard ratio [AHR], 2.32 [95% CI, 1.92-2.81]) and additional CCCs not associated with SNI (AHR, 1.70 [95% CI, 1.43-2.02]) (Figure 3).

### Short-Term Survival Based on PICU Episode Characteristics

Across all conditions, 1-year survival was greater than 80% (Table 2; eFigure 2A-D in Supplement 1). Increasing numbers of preceding high-risk PICU-CIEs were associated with decreased survival in a dose-dependent manner; survival after the first discharge was 90% (95% CI, 89%-91%) compared with 81% (95% CI, 77%-86%) after the fourth (Table 2). Across standard-risk episodes, there was no difference in short-term 1-year survival between the first discharge (92% [95% CI, 91%-93%]) and the fourth discharge (94% [95% CI, 88%-100%]), although the second discharge had lower survival (85% [95% CI, 81%-89%]). Because of the small number of patients, we did not evaluate survival for 5 or more discharges.

**Table 2.** Short-Term Survival Conditioned on Number and Intensity of PICU-CIEs in the Preceding Year

Episodes within preceding year	After standard-risk PICU-CIEs		After high-risk PICU-CIEs^a^	
	Episodes at risk	1-y Survival (95% CI)	Episodes at risk	1-y survival (95% CI)
First discharge	2557	0.92 (0.91-0.93)	3155	0.90 (0.89-0.91)
Second discharge	333	0.85 (0.81-0.89)	1029	0.85 (0.81-0.89)
Third discharge	125	0.89 (0.84-0.95)	504	0.84 (0.80-0.87)
Fourth discharge	68	0.94 (0.88-1.00)	279	0.81 (0.77-0.86)

Abbreviation: PICU-CIEs, pediatric intensive care unit critical illness episodes.

^a^ An episode was classified as high risk if it or any of the earlier episodes in the preceding year included invasive mechanical ventilation or had a length of stay more than 15 days. The focus of this analysis is on the episode, not the child; a child with multiple critical illness episodes would contribute data for each individual episode.

## Discussion

In a large population-based sample, 17.2% of children with SNI had CIEs requiring PICU admission between 2002 and 2019. Among children who survived their first CIE, 79% were alive 15 years later; increased complexity was associated with higher mortality. One-year survival conditioned on the number and severity of episodes in the preceding year ranged from 81% to 94%. Among high-risk episodes, there was a modest dose-dependent association, with 1-year survival decreasing from 90% after the first episode to 81% after the fourth episode.

Our findings conform to other comparable studies. Reported 5-year survival rates of children with SNI after gastrostomy tube placement ranged from 76%^23^ to 86%.^24^ In a prior study of mortality after PICU admission, children with neurologic CCCs had 1-year survival ranging from 95% for children with a single discharge to 89% for those with multiple PICU discharges, as estimated from Kaplan-Meier plots.^2^ Most studies describing long-term survival focus on survival from birth, rather than from an event that can occur at any age, and thus are not comparable.

Our findings highlight the challenges in using population-level estimates to inform individual-level care. Severe neurologic impairment includes diagnoses with variable prognoses, so interpretation of findings for individuals requires nuance; our results must be contextualized based on a child’s specific clinical situation. For example, an infant with trisomy 18 has different projected survival than a teenager with cerebral palsy and recurrent PICU-CIEs. Furthermore, health administrative data provide no details about why clinical decisions were made. Decisions to withhold or withdraw life-sustaining technology are common before pediatric deaths—occurring prior to 40% of inpatient deaths^25^ and 70% of PICU deaths^26^—so mortality data do not indicate natural history. Finally, while prognosis is often part of advanced care planning conversations, survival estimates provide only one piece of information; our study gives no insight into quality of life or other important outcomes.^27^

### Strengths and Limitations

Our results should also be interpreted in light of the study’s strengths and limitations. Strengths include the validity and generalizability of our findings, which are enhanced by population-level data that capture cross-provincial hospitalizations and deaths occurring in any location (eg, home, hospital, or hospice). We identified the cohort using a published list of diagnosis codes associated with high-intensity neurologic impairment^15^; this list and analogous lists^28^ have been used in many prior studies about children with SNI^23,29,30,31^; in general, diagnostic code lists^14^ work well to describe population-level trends in health care use.^32^

However, several limitations are important to note. First, because diagnosis codes do not differentiate children based on functional level, this cohort likely includes children with SNI as well as those with milder impairments^14^ (eg, gross motor function classification system level 1 and level 2 cerebral palsy). However, inclusion in the cohort required 1 or more PICU-CIEs, which likely reduced the proportion of children with milder impairments. Furthermore, children with milder functional impairments would be less likely to have recurrent admissions, so their inclusion would likely exacerbate a dose-dependent mortality gradient between first and subsequent admissions. Second, there is likely a spectrum of mortality risks associated with a PICU episode, which we have treated as binary (high risk vs standard risk) and assessed with imperfect surrogates (length of stay and invasive mechanical ventilation). Some episodes were potentially misclassified as high risk if the reason for prolonged length of stay or mechanical ventilation was not severe illness (eg, delayed discharge for logistical reasons or planned ventilation between surgical stages); others were misclassified as standard risk because of earlier discharge to intermediate care units. Further evaluation of mortality using more specific markers for severe critical illness—such as admission type (medical or surgical; planned or unplanned), vasoactive medications, increased respiratory settings for children with tracheostomy, or acute critical illness diagnoses (eg, status epilepticus)—would be valuable. Similarly, mortality data for common subpopulations (eg, children with SNI and congenital heart disease) would also be helpful. Third, we made multiple methodological decisions that likely influenced the magnitude of the survival estimates. For example, we considered episodes less than 15 days among children with chronic ventilatory needs as standard risk, we assessed episodes as high risk if any preceding episodes were high risk, and we did not differentiate between static and progressive SNI diagnoses. Although specific estimates might vary based on analytic choices, because survival was more than 80% for all conditions, our interpretation—that most children with SNI discharged from the PICU, including those with multiple recent admissions, will survive at least 1 year—is likely robust. Fourth, our data could not differentiate anticipated from unanticipated deaths, so we could not assess the association of decisions to limit life-prolonging therapies with mortality. Fifth, this study did not assess the likelihood of future PICU admissions based on the number of recent PICU admissions. Because PICU admissions have significant quality-of-life implications,^33^ understanding this association is important for clinical decision-making and warrants further study.

## Conclusions

Critical illness episodes requiring PICU admission are common among children with SNI. Approximately 80% of children with SNI are alive 15 years after their first CIE. Short-term survival decreases with increased number of recent high-risk PICU-CIEs, from 90% after the first discharge to 81% after the fourth. Although these findings support the common hypothesis that recurrent PICU episodes are associated with increased mortality, the absolute difference in estimated survival (and the converse of estimated mortality) is modest. In the context of counseling parents regarding potential major decisions to transition from life-prolonging to comfort-focused therapy, how this finding is framed will be critical; while the risk of mortality nearly doubles (from 10% to 19%), the likelihood of survival (expressed as natural frequencies) decreases only slightly, from 9 of 10 patients to 8 of 10 patients. The modest association identified in this study challenges the common narrative that increasing frequency of recent PICU admissions indicates a substantially higher risk of subsequent mortality.

## Notes

Supplement 1. **eFigure 1.** Two-Year Survival of Children Younger Than and Older Than 1 Year With Severe Neurologic Impairment Following Their Initial Discharge From the Pediatric Intensive Care Unit **eFigure 2.** One-Year Survival After PICU Discharge Based on Number of PICU Admissions in the Preceding Year (1st to 4th Episode) Stratified by High Compared to Standard Risk **eAppendix 1.** Diagnosis and Intervention Codes Defining Children With Severe Neurologic Impairment **eAppendix 2.** Notes on Methodology
eReference.

Supplement 2.
Data Sharing Statement
