# Supplementary material for: Recurrent Intensive Care Episodes and Mortality Among Children With Severe Neurologic Impairment
Source: JAMA Netw Open. 2024 Mar 15;7(3):e241852. doi: 10.1001/jamanetworkopen.2024.1852 (PMC10943411; doi:10.1001/jamanetworkopen.2024.1852)
Supplement: Supplement 1. — eFigure 1. Two-Year Survival of Children Younger Than and Older Than 1 Year With Severe Neurologic Impairment Following Their Initial Discharge From the Pediatric Intensive Care Unit eFigure 2. One-Year Survival After PICU Discharge Based on Number of PICU Admissions in the Preceding Year (1st to 4th Episode) Stratified by High Compared to Standard Risk eAppendix 1. Diagnosis and Intervention Codes Defining Children With Severe Neurologic Impairment eAppendix 2. Notes on Methodology eReference. [file jamanetwopen-e241852-s001.pdf]

Supplementary Online Content

Nelson KE, Zhu J, Thomson J, et al. Recurrent intensive care episodes and mortality among children with severe neurologic impairment. *JAMA Netw Open.* 2024;7(3):e241852. doi:10.1001/jamanetworkopen.2024.1852

eFigure 1. Two-Year Survival of Children Younger Than and Older Than 1 Year With Severe Neurologic Impairment Following Their Initial Discharge From the Pediatric Intensive Care Unit
eFigure 2. One-Year Survival After PICU Discharge Based on Number of PICU Admissions in the Preceding Year (1st to 4th Episode) Stratified by High Compared to Standard Risk
eAppendix 1. Diagnosis and Intervention Codes Defining Children With Severe Neurologic Impairment
eAppendix 2. Notes on Methodology
eReference.

This supplementary material has been provided by the authors to give readers additional information about their work.

© 2024 Nelson KE et al. *JAMA Network Open.*

eFigure 1. Two-Year Survival of Children Younger Than and Older Than 1 Year With Severe Neurologic Impairment Following Their Initial Discharge From the Pediatric Intensive Care Unit. This figure shows the first two years of data from the long-term survival curve (Figure 2).

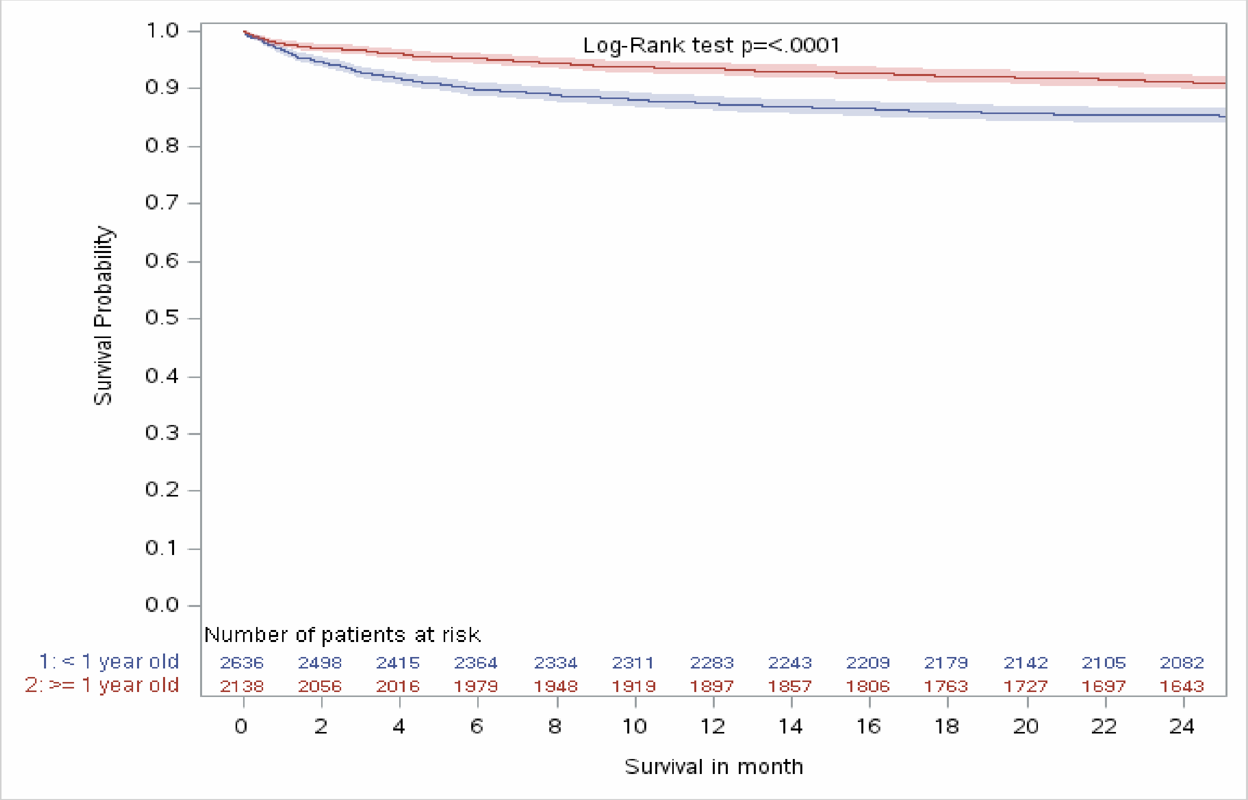

© 2024 Nelson KE et al. *JAMA Network Open.*

eFigures 2a-2d. One-Year Survival After PICU Discharge Based on Number of PICU Admissions In the Preceding Year (1st to 4th Episode) Stratified by High Compared to Standard Risk. An admission was considered high-risk if it or any of the earlier admissions within the year included invasive mechanical ventilation or length of stay >15 days.

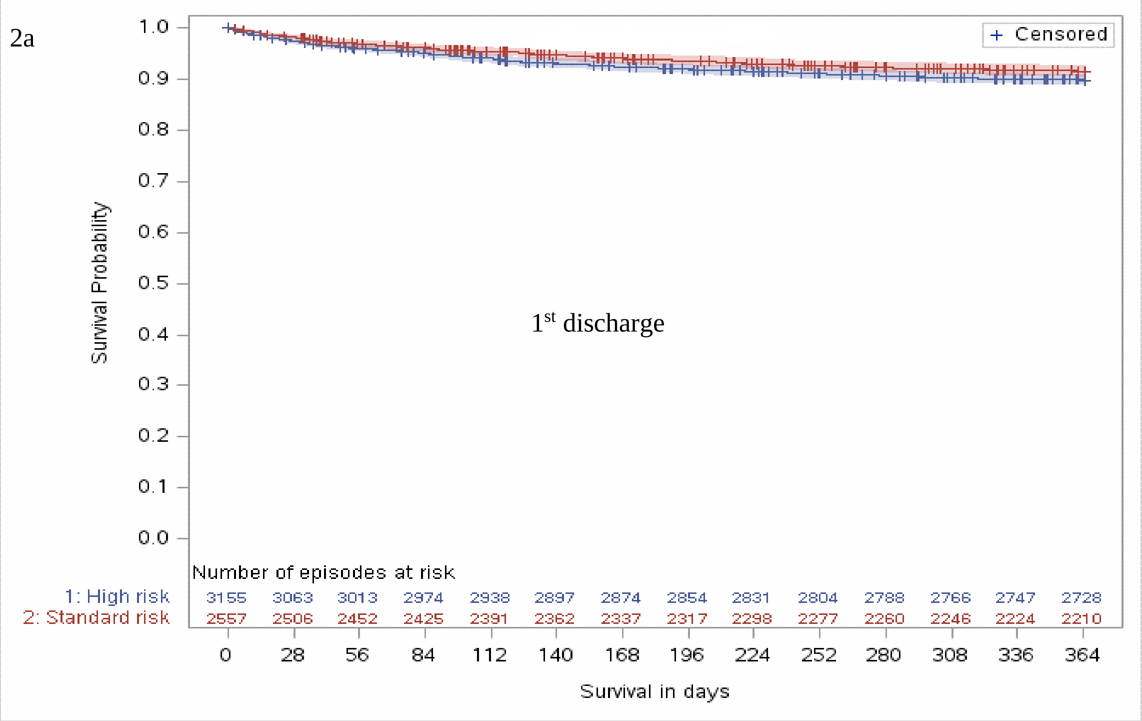

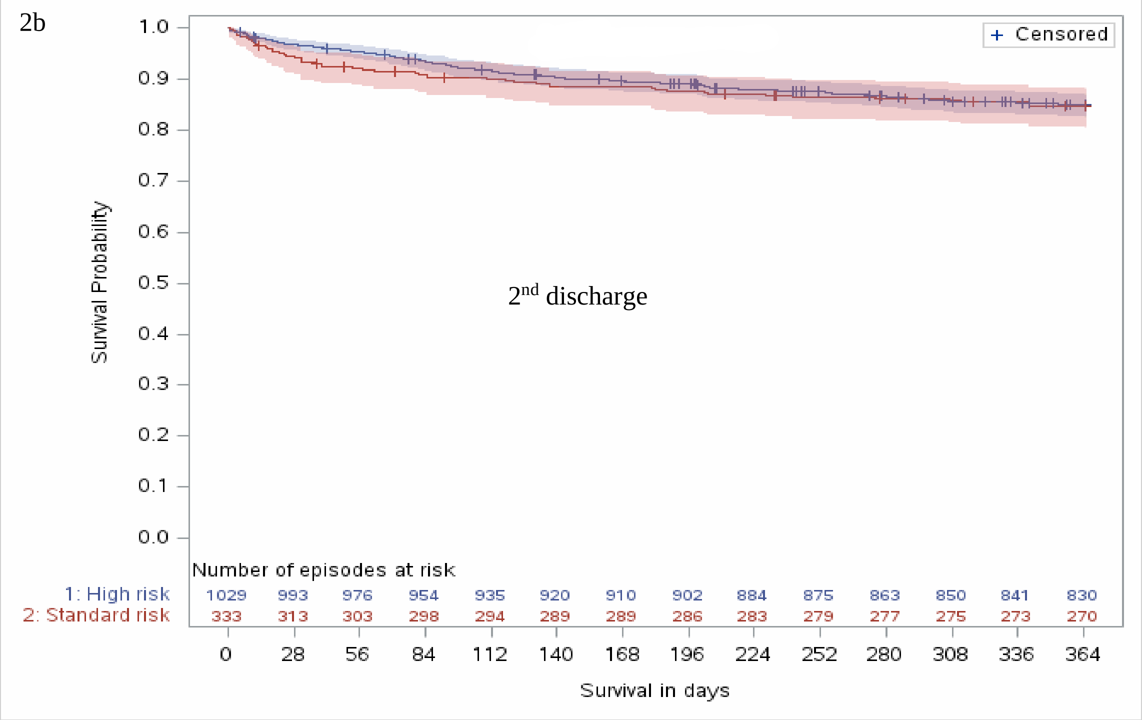

© 2024 Nelson KE et al. *JAMA Network Open.*

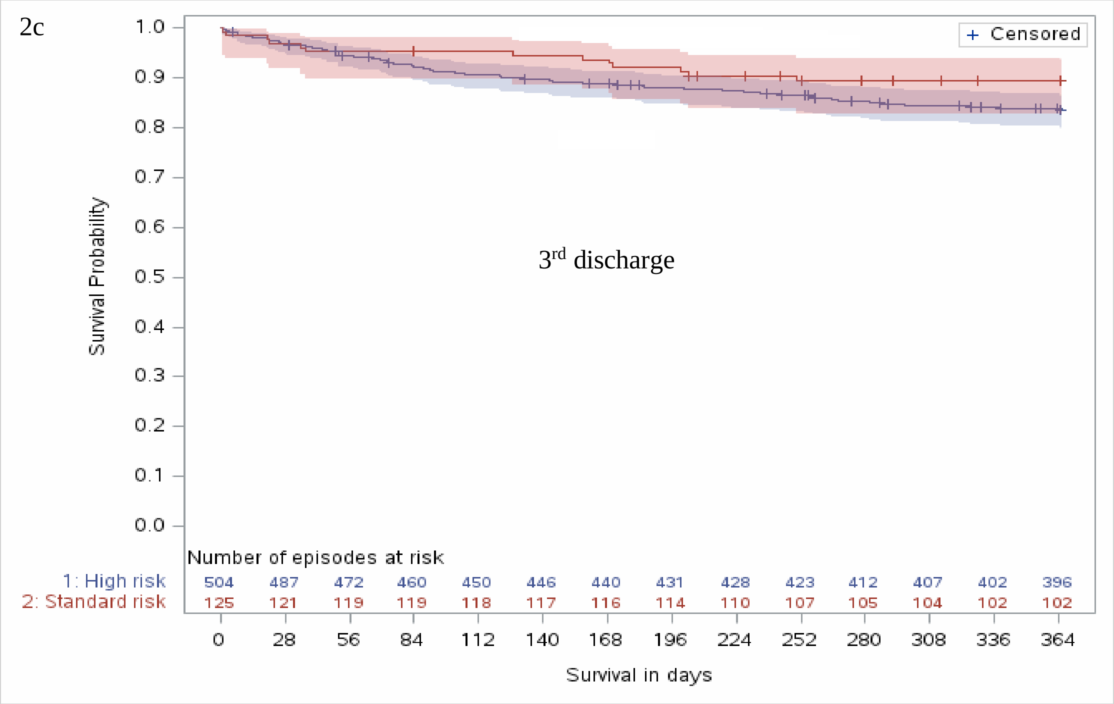

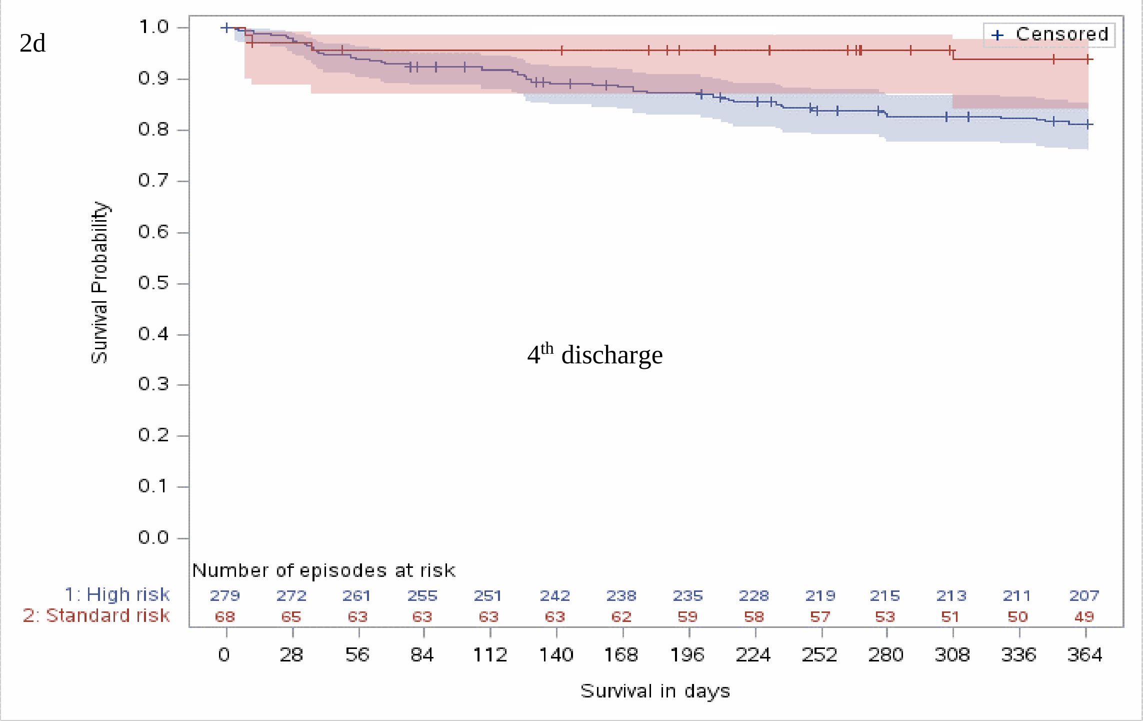

© 2024 Nelson KE et al. *JAMA Network Open.*

eAppendix 1. Diagnosis and Intervention Codes Defining Children With Severe Neurologic Impairment.[1] *ICD-10-CA: International Classification of Diseases, Version 10, Canada

Categories	ICD-10-CA*
Anatomic	G910, G911, G912, G913, G918, G919, G930, G935, G936, G9388, G939, G942, G948, G950, I780, Q000, Q001, Q002, Q010, Q011, Q012, Q018, Q019, Q02, Q030, Q031, Q038, Q039, Q040, Q041, Q042, Q043, Q044, Q045, Q046, Q048, Q049, Q050, Q051, Q052, Q053, Q054, Q055, Q056, Q057, Q058, Q059, Q060, Q061, Q062, Q063, Q064, Q068, Q069, Q070, Q078, Q079, Q761, Q850, Q851, Q858, Q859
Epilepsy	G400, G4000, G4001, G401, G4010, G4011, G402, G4020, G4021, G403, G4030, G4031, G404, G4040, G4041, G405, G4050, G4051, G4061, G4071, G408, G4080, G4081, G409, G4090, G4091
Genetic	D821, Q900, Q901, Q902, Q909, Q910, Q911, Q912, Q913, Q914, Q915, Q916, Q917, Q920, Q921, Q922, Q925, Q927, Q928, Q929, Q930, Q931, Q932, Q933, Q934, Q935, Q937, Q938, Q939, Q952, Q953, Q955, Q958, Q959, Q970, Q971, Q972, Q973, Q978, Q979, Q980, Q981, Q982, Q983, Q984, Q985, Q986, Q987, Q988, Q989, Q990, Q991, Q992
Metabolic	E700, E701, E702, E713, E720, E721, E722, E723, E724, E725, E728, E729, E740, E744, E748, E749, E755, E756, E770, E771, E778, E779, E786, E788, E789, E790, E791, E798, E799, E830, E851
Peripheral	A800, A801, A802, A803, A804, A809, B91, G041, G120, G121, G122, G128, G129, G130, G14, G35, G360, G361, G368, G369, G370, G371, G372, G373, G374, G375, G378, G379, G540, G541, G600, G601, G602, G603, G608, G609, G611, G618, G619, G628, G629, G700, G701, G702, G708, G709, G710, G711, G712, G713, G718, G719, G723, G731, G733, G737, G8100, G8101, G8109, G8110, G8111, G8119, G8190, G8191, G8199, G82011, G82012, G82013, G82021, G82022, G82023, G82091, G82092, G82093, G82111, G82112, G82113, G82121, G82122, G82123, G82191, G82192, G82193, G82211, G82212, G82213, G82221, G82222, G82223, G82291, G82292, G82293, G82310, G82311, G82320, G82321, G82390, G82391, G82410, G82411, G82420, G82421, G82490, G82491, G82510, G82511, G82520, G82521, G82590, G82591, G830, G831, G8320, G8321, G8322, G833, G834, G835, G838, G839, G951, P941, P942, T911, T913
Progressive/movement	A810, A811, A812, A818, E750, E750, E751, E751, E752, E752, E753, E754, E754, E760, E761, E762, E763, E768, E769, F842, G10, G110, G111, G112, G113, G114, G118, G119, G131, G132, G138, G20, G211, G212, G213, G214, G218, G219, G230, G231, G232, G238, G239, G241, G242, G248, G255, G258, G259, G26, G310, G3100, G3102, G311, G312, G318, G319, G320, G328, G901, G903
Static	A500, A504, A505, A506, A507, G800, G801, G802, G803, G804, G808, G809, G92, G931, G934, G937, G9381, P350, P351, P352, P570, P578, P579, P910, P911, P912, P915, P916, R4020, R4029, T903
Stroke/hemorrhage	G450, G451, G452, G453, G454, G458, G459, G460, G461, G462, G463, G464, G465, G466, G467, G468, I630, I631, I632, I633, I634, I635, I636, I638, I639, I64, I670, I671, I672, I673, I674, I675, I676, I677, I678, I679, I680, I690, I691, I692, I693, I694, I698, I720, I726, I728, S06140, S06141, S06240, S06241, S06340, S06341, S06440, S06441, S06540, S06541, S06640, S06641, S06840, S06841, S06940, S06941, T901, T905, T908, T909

© 2024 Nelson KE et al. *JAMA Network Open.*

eAppendix 2. Notes on Methodology

<u>Specific Health Administrative Data Code Definitions</u>

Pediatric Intensive Care Unit (PICU) admissions: PICU admissions were identified by special unit code (SCU=70), which is specific to pediatric intensive care and distinct from the codes used to indicate neonatal intensive care.

Post-procedural PICU admissions (for exclusion): We ascertained admission dates for all PICU admissions with length of stay (LOS) ≤2 days. We then identified procedures requiring anesthesia (surgical fee codes beginning with *D, E, J0, M, N, R, S, T, Z* and ending with the "C" suffix that indicates anesthesia) with procedure dates within 1 day of the PICU admission date.

Invasive mechanical ventilation: Canadian Classification of Intervention codes *1GZ31CAEP, 1GZ31CAND, 1GZ31CAPK* on the hospitalization record or a billing code for intubation (G211) with date of service occurring within 2 days of the critical illness episode dates (indicated by PICU admission and discharge dates). We allowed this date flexibility because intensive care is required for all invasively ventilated patients, so date misalignment most likely reflects clerical discrepancies between the hospitalization and physician billing datasets.

Emigration: When Ontario residents move out of province, they lose eligibility for provincial insurance. We censored children's data from the date that they became ineligible for the Ontario health insurance plan (OHIP).

<u>Analysis</u>

Because a child could have PICU episodes spaced >1 year apart, they could potentially be resampled for specific conditions (e.g., a child with two high-risk PICU admissions in 2014 and 2018 would contribute 1 year of survival data for each episode to the High-Risk "First Discharge" conditional survival curve). We used robust standard errors for conditions in which children had multiple entries, which only occurred for the Standard and High-Risk "First Discharge" curves.

© 2024 Nelson KE et al. *JAMA Network Open.*

eReference.

1. Nelson KE, Chakravarti V, Diskin C, et al. Validation of Neurologic Impairment Diagnosis Codes as Signifying Documented Functional Impairment in Hospitalized Children. *Academic Pediatrics*. Published online July 2021:S1876285921003764. doi:10.1016/j.acap.2021.07.014

© 2024 Nelson KE et al. *JAMA Network Open.*
